# Retrieval-Based Learning: Positive Effects of Retrieval Practice in Elementary School Children

**DOI:** 10.3389/fpsyg.2016.00350

**Published:** 2016-03-11

**Authors:** Jeffrey D. Karpicke, Janell R. Blunt, Megan A. Smith

**Affiliations:** ^1^Department of Psychological Sciences, Purdue University, West LafayetteIN, USA; ^2^Department of Psychology, Rhode Island College, ProvidenceRI, USA

**Keywords:** retrieval practice, learning, memory, individual differences, children

## Abstract

A wealth of research has demonstrated that practicing retrieval is a powerful way to enhance learning. However, nearly all prior research has examined retrieval practice with college students. Little is known about retrieval practice in children, and even less is known about possible individual differences in retrieval practice. In three experiments, 88 children (mean age 10 years) studied a list of words and either restudied the items or practiced retrieving them. They then took a final free recall test (Experiments 1 and 2) or recognition test (Experiment 3). In all experiments, children showed robust retrieval practice effects. Although a range of individual differences in reading comprehension and processing speed were observed among these children, the benefits of retrieval practice were independent of these factors. The results contribute to the growing body of research supporting the mnemonic benefits of retrieval practice and provide preliminary evidence that practicing retrieval may be an effective learning strategy for children with varying levels of reading comprehension and processing speed.

## Introduction

Learning is often thought to occur primarily when people study and encode new material, while retrieval is often considered only a neutral means of assessing knowledge. In contrast to the latter assumption, a wealth of research has demonstrated the powerful benefits of retrieval with a variety of materials such as word lists (e.g., Karpicke and Roediger, 2007, 2008; Pyc and Rawson, 2009, 2010) and educationally relevant texts (e.g., Roediger and Karpicke, 2006b; McDaniel et al., 2009; Karpicke and Roediger, 2010; Karpicke and Blunt, 2011). The vast majority of work on retrieval-based learning has been done with college-aged students. Few studies have examined retrieval practice effects in children, and even fewer have examined possible individual differences in retrieval-based learning (see Dunlosky et al., 2013). The purpose of the present research was to examine the effectiveness of retrieval-based learning strategies with elementary school-aged children and to explore individual differences in retrieval practice effects.

A great deal of prior research on cognitive strategies has been carried out with middle- and high-school students, whereas less work has been done with elementary school children (Pressley and Hilden, 2006). Nevertheless, the elementary school years represent a critical time in children’s development. At these ages, children are in a transitional phase in which they have learned to read and are increasingly “reading to learn.” Children are now expected to begin implementing learning strategies on their own, even though they may have difficulties executing effective strategies (Pressley and Harris, 2006). Further, research on developmental trajectories of episodic memory in children suggest that development of episodic memory, specifically the ability to form temporal associations, increases dramatically between the ages of 9 and 10 (Guillery-Girard et al., 2013). Thus, for several reasons, it is important to examine whether cognitive strategies like retrieval practice that are effective with older students and adults are also effective with elementary school-age children.

There has been little prior work examining retrieval practice in young children. In one of the earliest experiments on retrieval practice, Gates (1917) found that children (ranging from 1st through 8th grade) benefitted from spending time practicing “recitation” of non-sense syllables and biographical facts (see Roediger and Karpicke, 2006a, for discussion of Gates’s research; see also Spitzer, 1939). Only recently, almost one hundred years later, has there been renewed interest in examining retrieval practice in children. For example, Marsh et al. (2012) found that children in 2nd grade benefitted from taking initial multiple-choice tests on general knowledge facts when they were given feedback on the tests, extending previous research with adults (see Roediger and Marsh, 2005). Bouwmeester and Verkoeijen (2011) found that recalling associatively related word lists improved children’s (ages 7–13) performance on a delayed recognition memory test, relative to not recalling the word lists initially. Other work has shown benefits of retrieval practice, when retrieval is followed by feedback, for learning vocabulary words (Fritz et al., 2007; Goossens et al., 2014a,b) and learning the locations of objects on maps (Rohrer et al., 2010) in preschool and elementary school children. Thus, there are reasons to expect that children may show retrieval practice effects, but the evidence base is somewhat limited.

To examine whether children benefit from the mnemonic effects of *retrieval*, *per se*, experiments must meet several criteria (see Karpicke et al., 2014b). First, a retrieval practice condition must be compared to a repeated study condition. This comparison is critical because without a repeated study condition, any advantage of retrieval practice may simply be due to re-exposure to the material, rather than to the act of retrieval itself. Second, retrieval practice conditions must examine retrieval without feedback or restudying. When retrieval conditions also involve restudy, any advantage may be due to processing during the restudy trials, rather than to the act of retrieval (Kornell et al., 2009; Grimaldi and Karpicke, 2012). Third, it follows from the previous point that retrieval practice conditions must ensure high levels of initial retrieval success. Successful retrieval is essential for retrieval practice; if students cannot recall much, there will likely be little or no benefit of retrieval practice (Karpicke et al., 2014a). For example, Karpicke et al. (2014a, Experiment 1) examined free recall – a retrieval-based learning strategy that has been shown time and again to be extremely effective for adult learners (e.g., Roediger and Karpicke, 2006b) – in 4th graders and found no benefit on learning measured 5 days later. Initial retrieval success in this experiment was very low (around 8%), and when initial success is low, retrieval does not lead to improved learning. At the same time, retrieval practice cannot be trivially easy. For instance, massed repeated retrieval leads to high levels of initial success but very poor long-term retention (Carpenter and DeLosh, 2005; Karpicke and Bauernschmidt, 2011). This leads to the fourth condition, which is that retrieval practice must require learners to think back to and reinstate a prior episodic context (Karpicke and Zaromb, 2010; Lehman et al., 2014). In prior research on retrieval practice with children, many experiments did not include a restudy control condition (e.g., Marsh et al., 2012) or used retrieval practice conditions that involved feedback or restudying (Fritz et al., 2007; Rohrer et al., 2010; Goossens et al., 2014a,b). Few experiments examining retrieval practice with elementary school children have met the four criteria described here. The present experiments were designed with these criteria in mind.

The primary goals of the present experiments were to examine retrieval practice in children and to develop a method that promoted high levels of initial retrieval success while also requiring subjects to recollect a prior study context. Importantly, we did not use feedback to ensure any learning benefits found were direct benefits of retrieval practice and not indirect effects (Grimaldi and Karpicke, 2012). The procedure we used was adapted from Karpicke and Zaromb (2010) and was tailored for use with elementary school children. In an initial study phase (Phase 1), children studied a list of unrelated target words (e.g., *banana, football*). In Phase 2, the children either restudied the targets paired with category cues (e.g., *fruit: banana; sport: football*) or retrieved the targets, when given the cues and first two letters of the targets (e.g., *fruit: ba___; sport: fo____*). In Phase 3, after a brief delay, the children took a final free recall test (Experiments 1 and 2) or recognition test (Experiment 3).

A second goal of the current experiments was exploratory in nature and was concerned with examining individual differences in retrieval practice. Despite a wealth of research on retrieval practice effects, surprisingly little research has explored individual differences (Dunlosky et al., 2013), and to our knowledge, no research has been conducted to examine possible individual differences in retrieval-based learning with elementary school children. The current experiments examined two measures that have been linked to academic performance and are easily administered in a classroom setting: reading comprehension and processing speed. Children who perform better on measures of reading comprehension and processing speed are thought to create more elaborate mental models relative to children who score lower on these measures (Gernsbacher et al., 1990; McNamara and Magliano, 2009). Thus, one possibility is that children who score lower on these measures may show smaller retrieval practice effects, if retrieval practice depends primarily on such processes. Alternatively, if retrieval practice relies on recovering prior episodic contexts (Karpicke et al., 2014b), then the mnemonic effects of retrieval may be independent of individual differences in reading comprehension or processing speed.

Reading comprehension and processing speed were assessed with the Maze test (Fuchs and Fuchs, 1992) and the Cross-Out task from the Woodcock and Johnson (1989) Tests of Cognitive Ability (see Kail and Hall, 1994), respectively. In the Maze test, children read prose passages in which every seventh word in the passage was deleted and replaced with three possible word choices, only one of which was correct (e.g., *He was late, so he* ***map/see/ran*** *to catch the bus*). Children were instructed to select the word that correctly completed the sentence. In the Cross-Out task, rows of geometric figures were presented and children were instructed to cross out all figures in each row that were identical to the target figure in that row.

The three experiments reported below were part of a larger series of experiments conducted with 4th grade children throughout the 2012–2013 school year. The general procedure was similar in all three experiments, so the methods and results are described together. Experiment 1 examined the effect of retrieval practice on a final free recall test. Experiment 2 was aimed at replicating Experiment 1 with a different group of 4th grade children and a new set of materials. Experiment 3 examined the effects of retrieval practice on a final yes/no recognition memory test.

## Experiments 1–3

### Method

#### Subjects

A total of 88 children participated in the three experiments. They were recruited from four fourth-grade general education classrooms in a public school in Indianapolis, Indiana. The mean age of the children was 10.0 years (*SD* = 0.5, range = 9.2–12.0). The school’s total student population was 52% African American, 27% Hispanic, 14% Caucasian, and 7% other races/ethnicities. In exchange for participating in the experiment, children received a gift card to use at their school’s semiannual book fair. The experiments were approved by the Purdue University Institutional Review Board (IRB) and followed all APA guidelines for the ethical treatment of human subjects. Parental consent and student assent was received for each child.

Forty children from two classrooms participated in Experiment 1, which occurred in the fall semester. Forty children from two different classrooms participated in Experiment 2 in the spring semester. Forty-two children from the two classrooms in Experiment 2 also participated in Experiment 3, which occurred in the fall semester but is presented third for expositional purposes. Thirty-four children participated in both Experiments 2 and 3, which used different sets of materials and different final test formats. Six children participated in Experiment 2 but not Experiment 3, and eight children participated in Experiment 3 but not Experiment 2. This was largely due to fluctuation of children in and out of the school across semesters.

#### Materials

Two lists of 24 target words were used in the three experiments. The same word list was used in Experiments 1 and 3, because different children participated in the two experiments. Each target word had a unique category label (e.g., the target words *banana* and *football* had the category labels *fruit* and *sport*, respectively) and was three to eight letters in length. Most items were selected from the category norms of Posnansky (1978), and a few were generated by the experimenters. The average age of acquisition for the words was 4.7 years (Kuperman et al., 2012). Each list was divided into two sets of 12 items. The two sets were equated in terms of word length (Experiments 1 and 3, 5.3 and 5.2; Experiment 2, 5.6 and 6.2), Kucera and Francis (1967) word frequency (Experiments 1 and 3, 30.1 and 30.5; Experiment 2, 22.4 and 26.5), and age of acquisition (Experiments 1 and 3, 4.3 and 4.4; Experiment 2, 5.0 and 5.0). The assignment of sets to condition was counterbalanced across children so that each set was presented in both conditions.

The recognition test in Experiment 3 comprised 36 words: the 24 target words and 12 new distracter words. The distracter words were drawn from the English Lexicon Project (Balota et al., 2007) such that the 12 distracters were matched with the total set of 24 target words in terms of word length, word frequency, and age of acquisition.

#### Design

Each experiment used a mixed-list, within-subject design. There were two conditions: retrieval practice and repeated study. During the critical retrieval practice and repeated study phase, all items were presented with a category cue. Half of the targets were presented as stems for the retrieval practice trials and half were presented intact for repeated study trials.

#### Procedure

The experiments were conducted in classrooms, and the children were tested as a group, with about 20 children per classroom, but were instructed to work independently. At the beginning of each experiment, the children were told that they would study a list of words for a later memory test. Each experiment consisted of three phases. In Phase 1, a list of 24 target words (e.g., *banana, football*) was presented on a projector screen at the front of the classroom. The words were shown simultaneously, in a single column, and the experimenter read each word out loud one at a time at the rate of approximately 2 s per word. After the experimenter had read the entire list, the children had an additional 1 min to study the words silently. The word list was then removed from the screen.

Phase 2 was the critical phase of each experiment in which the retrieval practice vs. repeated study manipulation occurred. The children were given a sheet that showed the 24 target words and category cues paired with each target. In the repeated study condition the items were shown intact (e.g., *fruit: banana; sport: football*); in the retrieval practice condition, the stems of the targets were shown (e.g., *fruit: ba___; sport: fo____*). The children were told to restudy the words that were intact and to recall a word that completed the stems. They made their responses by writing the words on the sheets. Children were given approximately 4 min to complete Phase 2.

In Experiments 1 and 2, Phase 3 involved a final free recall test. Children were given a sheet of lined paper and were asked to write down as many words as they could remember from the study list, in any order. Children were reminded that these were the words they had restudied or completed in the second part of the experiment, not the category clue words. The free recall test lasted approximately 4 min.

In Experiment 3, Phase 3 involved a final recognition test. Children were shown the original 24 target words and 12 distracter words simultaneously in a random order on a single sheet of paper. They were instructed to read each word and decide whether they had seen the word earlier in the experiment. The words “yes” and “no” were printed next to each test word, and the children circled “yes” to indicate that they had previously seen the word and “no” to indicate that they had not. The recognition test lasted approximately 4 min.

#### Maze Reading Comprehension Task

Children took the Maze reading comprehension test twice as a separate part of the experiment, once in the fall semester and once in the spring semester. The Maze task has high criterion validity (from 0.80 to 0.89; see Fuchs and Fuchs, 1992; Jenkins and Jewell, 1993) and high test–retest reliability (over 0.90; Fuchs and Fuchs, 1992). In this task, children read passages in which a word in each sentence was deleted and replaced with three choices, only one of which correctly completed the sentence (e.g., *He was late, so he* ***map/see/ran*** *to catch the bus*). Prior to the start of the task, the experimenter led the children through an example. They had 2.5 min to complete as many sentences as they could during the test.

#### Cross-Out Task

In the Cross-Out task, children viewed 30 rows of geometric figures. Each row contained a target figure on the left and 19 similar figures to the right. For example, one target was an oval with a square inside; the remaining 19 figures were ovals with various shapes inside (e.g., the identical square, a line, two dots, a triangle, or nothing). Within each row, 5 of the 19 figures were identical to the target. The children were instructed to place a line through the figures that were identical to the target figure on the left. They had 3 min to complete the task.

## Results and Discussion

Initial analyses showed no differences among the counterbalancing orders in each experiment, so the results have been collapsed across orders.

### Initial Retrieval Success

Importantly, the retrieval practice task led to high levels of initial retrieval success. The children were able to retrieve 87% (SEM = 3%), 75% (SEM = 3%), and 87% (SEM = 3%) of the target words in Experiments 1, 2, and 3, respectively.

### Final Free Recall (Experiments 1 and 2)

**FIGURE 1:**
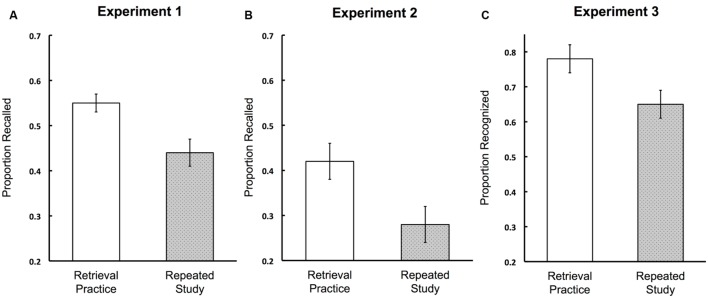
**Proportion of target words recalled on the final free recall test in Experiment 1 **(A)** and Experiment 2 **(B)**, and proportion of target words correctly recognized in Experiment 3 **(C)**.** Error bars represent standard error of the mean. Note that the scale is different in **(C)** than it is in **(A)** and **(B)**. In all three experiments, children recalled and recognized more words on the final test when they practiced retrieving the words relative to when they repeatedly studied them.

**Figure 1** shows the key results of Experiments 1 and 2: the proportion of words recalled on the final free recall tests. There was an advantage of retrieval practice over repeated study in Experiment 1 (0.55 vs. 0.44), *t*(39) = 3.02, *d* = 0.48, 95% CI [0.14, 0.80], and in Experiment 2 (0.42 vs. 0.28), *t*(39) = 3.57, *d* = 0.56 [0.22, 0.90]. Thus, Experiments 1 and 2 demonstrated robust positive effects of retrieval practice with elementary school children.

### Final Recognition (Experiment 3)

The critical result of Experiment 3 was performance on the final recognition test, shown on the right panel of **Figure 1.** Recognition was higher for retrieved vs. restudied words (0.77 vs. 0.66), *t*(41) = 4.17, *d* = 0.64 [0.31, 0.97]. False alarm rates were low (*M* = 0.13, *SE* = 0.03), consistent with prior work with similar methods (Karpicke and Zaromb, 2010). Like Experiments 1 and 2, Experiment 3 showed a robust retrieval practice effect in children.

### Individual Difference Measures

**FIGURE 2:**
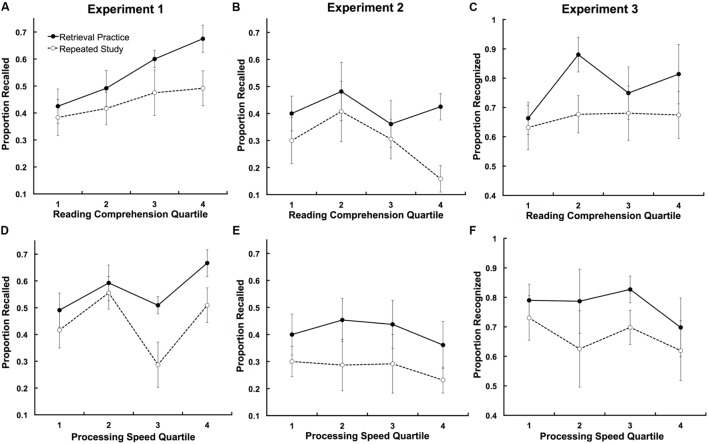
**Children’s retrieval practice and repeated study performance based on their reading comprehension and speed of processing.** For illustrative purposes, children were divided into quartiles, 1 representing the lowest 25% of scorers, and 4 representing the highest 25% of scorers. Reading comprehension **(A–C)** was measured by the Maze test, and speed of processing **(D–F)** was measured by the Cross-Out task from the Woodcock–Johnson Tests of Cognitive Ability. Across the three experiments, children consistently benefitted from retrieval practice over repeated study and this effect was approximately the same regardless of reading comprehension and speed of processing.

We assessed the role of individual differences in reading comprehension and processing speed by conducting ANCOVAs using these two measures as covariates, thus treating the individual difference measures as continuous variables (Poole and Kane, 2009). For illustrative purposes, **Figure 2** shows final recall and recognition performance by condition (retrieval practice vs. repeated study) for children classified into quartiles on the reading comprehension test (**Figures 2A–C**) and speed of processing test (**Figures 2D–F**). The figure shows that, in general, children showed positive effects of retrieval practice regardless of their performance on the reading comprehension or processing speed measures.

#### Maze Reading Comprehension Task

**FIGURE 3:**
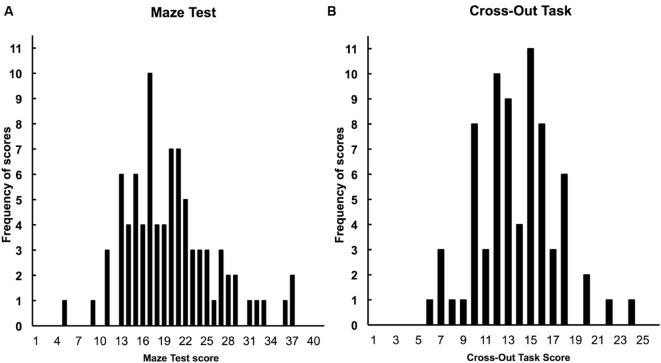
**Overall frequency distribution of Maze reading comprehension scores **(A**; skewness = 0.42; kurtosis = 0.93) and Cross-Out processing speed scores **(B**; skewness = 0.22, kurtosis = 0.32) combined for children from all experiments**.

The Maze test was scored by giving 1 point for each correctly circled answer. Responses left blank were counted as errors, and scoring was discontinued if three consecutive errors were made (Fuchs and Fuchs, 1992; Tilstra et al., 2009). The number of answers correctly circled within the designated amount of time was the child’s score. Two children did not complete the Maze task either in the fall or spring, 11 children completed the test only in the fall, and four children completed the test only in the spring. **Figure 3** shows the distribution of Maze test scores, which shows that the Maze scores were normally distributed and that there was a range of individual differences in reading comprehension within our student samples. Overall, reading comprehension improved from the fall to the spring (*M* = 18.0 vs. 22.7, *SD* = 6.0, 7.8), *t*(69) = 6.59, *d* = 1.11 [0.76, 1.47], and the average score in the spring was above the end-of-year performance goal (18) for typically developing children in fourth grade (Fuchs and Fuchs, 2011). The Maze tests from the fall and spring correlated at *r* = 0.67, and the pattern of results reported below was the same when either fall or spring Maze scores were used. Therefore, if children had two maze scores (both fall and spring, *n* = 70 children), the scores were averaged; if children only completed one Maze test, the single score from the completed test was used. Scores were then converted to *z*-scores. All scores were included in the distribution shown in **Figure 3.**

#### Cross-Out Task

To score the Cross-Out processing speed task, children were given 1 point for each correctly completed row. Rows that did not have all five target figures crossed out or rows that had incorrect figures crossed out were not counted as correct (see Kail and Hall, 1994).

The processing speed test was administered in the spring semester. The distribution of scores from children across all three experiments is presented in **Figure 3.** Sixteen children did not complete the processing speed task (4 children from Experiment 1; 5 children from Experiment 2; and 10 children from Experiment 3, including 3 who participated in Experiment 2) and were therefore excluded from the following analyses. The average score was 14.7 (*SD* = 0.5). **Figure 3** shows that, as was the case with the Maze test scores, the speed of processing scores were normally distributed, and there was a range of individual differences in speed of processing scores within our student samples.

#### Individual Differences: Experiment 1

Two repeated measures ANCOVAs were carried out with condition (retrieval vs. restudy) as the independent variable, recall performance as the dependent variable, and individual difference measure (either reading comprehension or processing speed) as the covariate. Scores on the reading comprehension and speed of processing tests were analyzed as continuous variables in the ANCOVAs (the data were binned into quartiles in **Figure 2** simply to make the figure clearer). The analyses showed a main effect of reading comprehension, *F*(1,38) = 6.38, $\eta_{p}^{2}$ = 0.14, but not processing speed, *F*(1,34) = 0.70, $\eta_{p}^{2}$ = 0.02. Children with higher reading comprehension scores performed better on the final test, whereas final test performance did not differ as a function of processing speed. There were no reading comprehension × condition or processing speed × condition interactions, *F*(1,38) = 1.21, $\eta_{p}^{2}$ = 0.03; *F*(1,34) = 1.28, $\eta_{p}^{2}$ = 0.04. Importantly, the ANCOVAs showed that when reading comprehension and processing speed were entered as covariates, there were still robust effects of retrieval practice in each analysis, *F*(1,38) = 8.85, $\eta_{p}^{2}$ = 0.19, and *F*(1,34) = 11.14, $\eta_{p}^{2}$ = 0.25, respectively^1^. Overall, the results indicate that the benefit of retrieval practice over repeated study was approximately equivalent for children at all levels of reading comprehension and processing speed.

#### Individual Differences: Experiment 2

The ANCOVAs carried out for Experiment 1 were also performed on the data from Experiment 2, and in general, the patterns of results were the same. The ANCOVAs showed no main effects of reading comprehension or processing speed, *F*(1,36) = 0.69, $\eta_{p}^{2}$ = 0.04, and *F*(1,33) = 1.31, $\eta_{p}^{2}$ = 0.04, respectively. As in Experiment 1, there were main effects of retrieval practice, *F*(1,36) = 10.05, $\eta_{p}^{2}$ = 0.22, and *F*(1,33) = 11.59, $\eta_{p}^{2}$ = 0.26. There were no reading comprehension X condition or processing speed × condition interactions, *F*(1,36) = 0.69, $\eta_{p}^{2}$ = 0.04, and *F*(1,33) = 0.88, $\eta_{p}^{2}$ = 0.03, respectively.

#### Individual Differences: Experiment 3

The ANCOVAs were performed on the data from Experiment 3, and the results were the same as those in the previous experiments. Once again, the ANCOVAs showed no main effects of reading comprehension or processing speed, *F*(1,38) = 0.18, $\eta_{p}^{2}$ = 0.01; *F*(1,30) = 0.25, $\eta_{p}^{2}$ = 0.01. There were main effects of retrieval practice, *F*(1,38) = 15.63, $\eta_{p}^{2}$ = 0.29, and *F*(1,30) = 10.30, $\eta_{p}^{2}$ = 0.26, and no reading comprehension × condition or processing speed × condition interactions, *F*(1,38) = 0.00, $\eta_{p}^{2}$ = 0.00; *F*(1,30) = 0.40, $\eta_{p}^{2}$ = 0.02.

## General Discussion

The purpose of the present experiments was to examine the effectiveness of retrieval practice with elementary school children. Taken collectively, the combination of results from these three experiments suggests that the retrieval practice produces robust learning with elementary school children and that these effects do not appear to depend on individual differences in reading comprehension or processing speed.

Practicing retrieval enhanced learning in elementary school children on final free recall and recognition tests. These results suggest that children who are in a critical transitional phase in formal education (fourth grade, roughly age 10) show the kinds of robust mnemonic benefits of retrieval practice that older children (e.g., Roediger et al., 2011) and college students show. A critical factor in retrieval-based learning is initial retrieval success. Unless retrieval activities are designed to ensure initial retrieval success, children will not benefit from retrieval practice (Karpicke et al., 2014a). Previous experiments examining the effectiveness of retrieval practice in children typically included feedback (e.g., Fritz et al., 2007; Rohrer et al., 2010; Goossens et al., 2014a), and while feedback unquestionably improves learning, the provision of feedback makes it impossible to examine the direct mnemonic effects of retrieval, *per se* (Roediger and Karpicke, 2006a). The present experiments used a method that boosted initial retrieval success by providing cues during initial retrieval but also required children to think back to the original study list, following Karpicke and Zaromb (2010). No feedback was given, so the present effects were entirely driven by direct benefits of retrieval. The results extend the evidence for the effectiveness of retrieval practice in children by showing direct benefits of retrieval practice on retention.

The effects of retrieval practice were largely the same for children with varying levels of reading comprehension and processing speed scores. Collectively, the preliminary results from the current experiments provide no evidence that retrieval practice was moderated by these two factors. The present findings are similar to findings by Goossens et al. (2014a). They found benefits of retrieval practice with feedback in elementary school children who learned vocabulary words. Goossens et al. (2014a) reported that individual differences on a standardized test of children’s vocabulary size test did not interact with the size of the retrieval practice effect. Just as the benefits of retrieval practice were similar for children with high or low vocabulary sizes, the present results suggest the benefits are similar regardless of whether children have high or low reading comprehension and processing speed scores. Further research is needed to directly assess the extent to which the benefits of retrieval practice depend on the characteristics of individual learners. It is possible that with a wider range of individual difference scores and additional children, there may be individual difference factors that matter for retrieval practice effects.

The present experiments were not designed to delineate among theories of retrieval practice, but the individual difference results may have theoretical implications. Current theories of reading comprehension suggest that children with higher reading comprehension abilities are better at creating more enriched and elaborate mental models of the material they are learning (McNamara and Magliano, 2009). These children may be better in general at forming elaborative connections and semantic associations among materials. One current theory of retrieval-based learning is the elaborative retrieval account, which proposes that semantic elaboration is the basis of retrieval practice effects (see Carpenter, 2011). If children with higher reading comprehension scores are better at forming elaborations, then these children might show greater retrieval practice effects than children with lower reading comprehension scores, who may show little or no benefit of retrieval. In contrast, the present experiments suggest that the benefits of retrieval practice are independent of children’s reading comprehension abilities. The present experiments were not designed to test the elaborative retrieval account directly, but the results showed benefits of retrieval practice independent of individual differences in reading comprehension, which may be a proxy for the ability to form elaborations.

The present results are consistent with an episodic context account of retrieval-based learning (Karpicke et al., 2014b; Lehman et al., 2014), although the experiments were not designed to test that account directly. The episodic context account proposes that retrieval requires people to reinstate a prior learning context. When retrieval is successful, the context associated with an item is updated to include features of the retrieved and present contexts. The refined context representation, which would continue to be updated with additional repeated retrieval, enhances the likelihood of subsequent retrieval on a criterial test. The present results indicate that children were capable of engaging in the type of episodic context retrieval necessary to produce retrieval practice effects (Guillery-Girard et al., 2013) and that the benefits of retrieval practice were independent of measures of semantic/elaborative processing and speed of processing.

The key finding from the present experiments was that retrieval practice enhanced retention relative to repeated study with elementary school children and that this effect does not appear to depend on reading comprehension or processing speed. The present results show that when children are able to successfully retrieve initially during retrieval practice, they benefit from retrieval practice.

## Author Contributions

All three authors designed the experiments, collected and analyzed the data, and wrote this report.

## Conflict of Interest Statement

The authors declare that the research was conducted in the absence of any commercial or financial relationships that could be construed as a potential conflict of interest.

## Footnotes

1 We also conducted a separate ANCOVA with age as a covariate. Across all experiments, there was no main effect of age, indicating that children within the age range of our sample performed approximately equally on the final test. There was a main effect of retrieval practice, and no age × condition interaction, indicating that retrieval practice produced robust effects regardless of children’s ages.
